# Rapid and accurate interpretation of dengue diagnostics in the context of dengue vaccination implementation: Viewpoints and guidelines issued from an experts group consultation

**DOI:** 10.1371/journal.pntd.0005719

**Published:** 2017-09-07

**Authors:** Elizabeth A. Hunsperger, Claudia N. Duarte dos Santos, Huong Thi Que Vu, Sutee Yoksan, Vincent Deubel

**Affiliations:** 1 Centers for Disease Control, Global Disease Detection Regional Center, Nairobi, Kenya; 2 Laboratory of Molecular Virology, Institute Carlos Chagas/Fiocruz, Curitiba, Brazil; 3 Arbovirus Laboratory, Pasteur Institute of Ho Chi Minh City, Ho Chi Minh City, Vietnam; 4 Center for Vaccine Development, Mahidol University, Bangkok, Thailand; 5 Pasteur Institute, Paris, France; Centers for Disease Control and Prevention, UNITED STATES

## Introduction

There are 4 serologically distinct dengue viruses (DENVs), which contemporarily cocirculate in a majority of countries of the tropical and subtropical belt in Latin America, Asia-Pacific, and Africa [1]. DENVs are members of the *Flavivirus* genus, related to other medically important mosquito-borne viruses such as yellow fever virus (YFV), Japanese encephalitis virus (JEV), and Zika virus (ZIKV). A chimeric YF-DEN tetravalent dengue vaccine CYD-TDV (Dengvaxia) has now been licensed in 13 countries [2]. A phase IIb trial in Thailand and 2 phase III large-scale efficacy trials in 10 countries in Asia and Latin America have been conducted [2]. The vaccine clinical monitoring and careful diagnosis of the vaccinated and unvaccinated populations enrolled in these studies have provided valuable information that is useful for the later stage of vaccination implementation and its monitoring [2]. A recent study of the validity of the serological diagnosis of dengue immunoglobulin G (IgG) and immunoglobulin M (IgM) by ELISA according to WHO recommendations [3] in acute febrile and in convalescent individuals vaccinated and unvaccinated (Placebo), and virologically confirmed or not, showed a high sensitivity (97.1%) but a low specificity (85.1%) in the IgM ELISA due to the presence of residual IgM from previous vaccination or subclinical undetected dengue, which may introduce a diagnostic bias [4]. The proportion of false positives IgM in the CYD-TDV vaccinated group (17.4%) was higher than in the control group (10.1%), particularly within the 2 months following vaccination. Therefore, it was necessary to review and redefine a reliable diagnostic testing algorithm in light of false dengue positives, which would be used by healthcare professionals to confirm suspected dengue cases in vaccinees and to evaluate the effectiveness of the CYD-TDV vaccine in the vaccinated populations. Here, a group of experts in the diagnosis and monitoring of DENV and other flaviviruses has considered key questions regarding sensitive and specific dengue diagnostics and their application for dengue surveillance in countries where the CYD-TDV vaccine has been implemented.

## Dengue diagnostics and their limitations

There are several virological and serological approaches to diagnose DENV in blood with different sensitivities and specificities [3,5–7]. The advantages and limitations of the different techniques for the diagnosis of dengue have been described elsewhere [3,5]. Diagnostic techniques targeting viral biomarkers are generally positive in the first 5 days post onset (DPO) and include virus isolation in mosquito or mammalian cell cultures, detection of viral RNA by reverse transcriptase-polymerase chain reaction (RT-PCR), and immunodiagnostic tests that capture soluble nonstructural protein 1 (NS1) antigen. The gene amplification technique is faster and more sensitive than cell culture, especially in its version "Real-Time" RT-PCR, but it remains complex, expensive, and time consuming. Therefore, it is not adapted for large dengue surveillance and vaccine evaluation process. NS1 is an important biomarker for early diagnosis during the acute phase of dengue infection paralleling virus replication for 1 to 4 DPO [7–9]. The sensitivity of the dengue NS1 antigen ELISA (NS1 ELISA) can vary according to the viral serotypes [7–9]. Companies use different monoclonal antibodies for NS1 antigen capture, which may affect its sensitivity depending on DENV serotypes [7–9]. NS1 titers are generally lower in secondary versus primary dengue infection due to immune complex formation that occurs in secondary infections [5,8]. These different factors may lead to suboptimal ELISA results of NS1 (Table 1). NS1 ELISA has the advantages of rapidity, being easy to perform, and being cost-effective [7].

**Table 1:** Results of dengue NS1 ELISA and IgM ELISA depending on time points of the blood collection after the onset of symptoms, the patient natural history and serological status, or the cause of the disease.

	Time of sample collection (DPO)					
Number of days	≤4		5 to 8		≥9	
Dengue NS1 antigen and IgM capture ELISA diagnostic tests	NS1	IgM	NS1	IgM	NS1	IgM
**Origin of the disease and serological status**						
Primary wild-type dengue infection	Pos^a^	Neg	Pos/Neg	Pos	Neg	Pos
Secondary wild-type dengue infection	Pos^a^	Pos/Neg	Pos/Neg	Pos	Neg	Pos
Dengue infection in CYD-TDV vaccinee	Pos	Pos/Neg	Pos/Neg	Pos	Neg	Pos
Undetected previous dengue infection^b^	Neg	Pos/Neg	Neg	Pos/Neg	Neg	Pos/Neg
Recent CYD-TDV vaccination^c^	Neg	Pos/Neg	Neg	Pos/Neg	Neg	Pos/Neg
Infection by a Flavivirus other than dengue^d^	Neg	Pos/Neg	Neg	Pos/Neg	Neg	Pos/Neg

^a^ Several factors may generate possible suboptimal detection of NS1 (see text)

^b^ Asymptomatic or missed dengue infection occurring less than 3 months before testing

^c^ Residual dengue IgM elicited by CYD-TDV may last at least 2 months [4]

^d^ Flavivirus cross-reactive antibodies (in secondary infection)

**Abbreviations:** DPO, days post onset; IgM, immunoglobin M; Pos, Positive result; Neg, Negative result; Pos/Neg, the sensitivity of the test, the time point of serum sampling, and the serological status of the patient may affect the result

The plaque reduction neutralization test (PRNT) and the hemagglutination inhibition (HI) test are 2 serological reference techniques for flavivirus antibody detection [3,5]. They require reference laboratories with a panel of different DENVs and flaviviruses and their derived antigens for more specific diagnosis [3]. Detection of anti-DENV IgG and/or anti-DENV IgM by ELISA is an easy-to-perform assay, which is not quantitative because it is usually performed on a single serum dilution [6]. During a primary infection with DENV, anti-DENV IgM antibodies appear ≥4 DPO and can persist beyond 3 months [6]; however, detection of anti-DENV IgM is indicative of recent infection but not necessarily a current infection [3,5,6]. During a secondary dengue infection, IgM rapidly increases early in the course of infection (Table 1) [6]. Similar to the PRNT or HI, IgM and IgG ELISAs can be cross-reactive in patients previously infected or vaccinated with another Flavivirus, thus the tests lack specificity within this family of viruses (Table 1) [3,5,6]. To assess an acute dengue infection, a second blood sample is taken at the convalescent phase to show the presence of IgM or an increase in IgM ELISA index value (ratio between the optical density [OD] of the sample and the OD of the cutoff), which is suggestive of an increase in IgM antibodies [3]. IgG ELISA test is usually not utilized for dengue diagnostic in endemic countries for DENV and other flaviviruses due to possible cross-reactivity. Moreover, a ≥4-fold dengue IgG ELISA value increase between acute and convalescent samples in the dengue-infected CYD-TDV group was observed in only 19.7% of cases compared to 35.5% of the control (placebo) group, ruling out such antibody rise as a viable diagnostic criterion [4]. The detection of soluble DENV NS1 antigen early in the course of infection combined with the detection of anti-DENV IgM increases the window for dengue diagnosis and provides an additive effect. (Table 1) [5,10–12]. The test’s value is optimized at specific time points: the optimal window for NS1 antigen detection is from 1 to 4 DPO and 5 to 14 DPO for IgM (Table 1) [7–12].

## Algorithm of diagnostics after CYD-TDV dengue vaccine implementation in countries with a high burden of dengue

**Fig 1:**
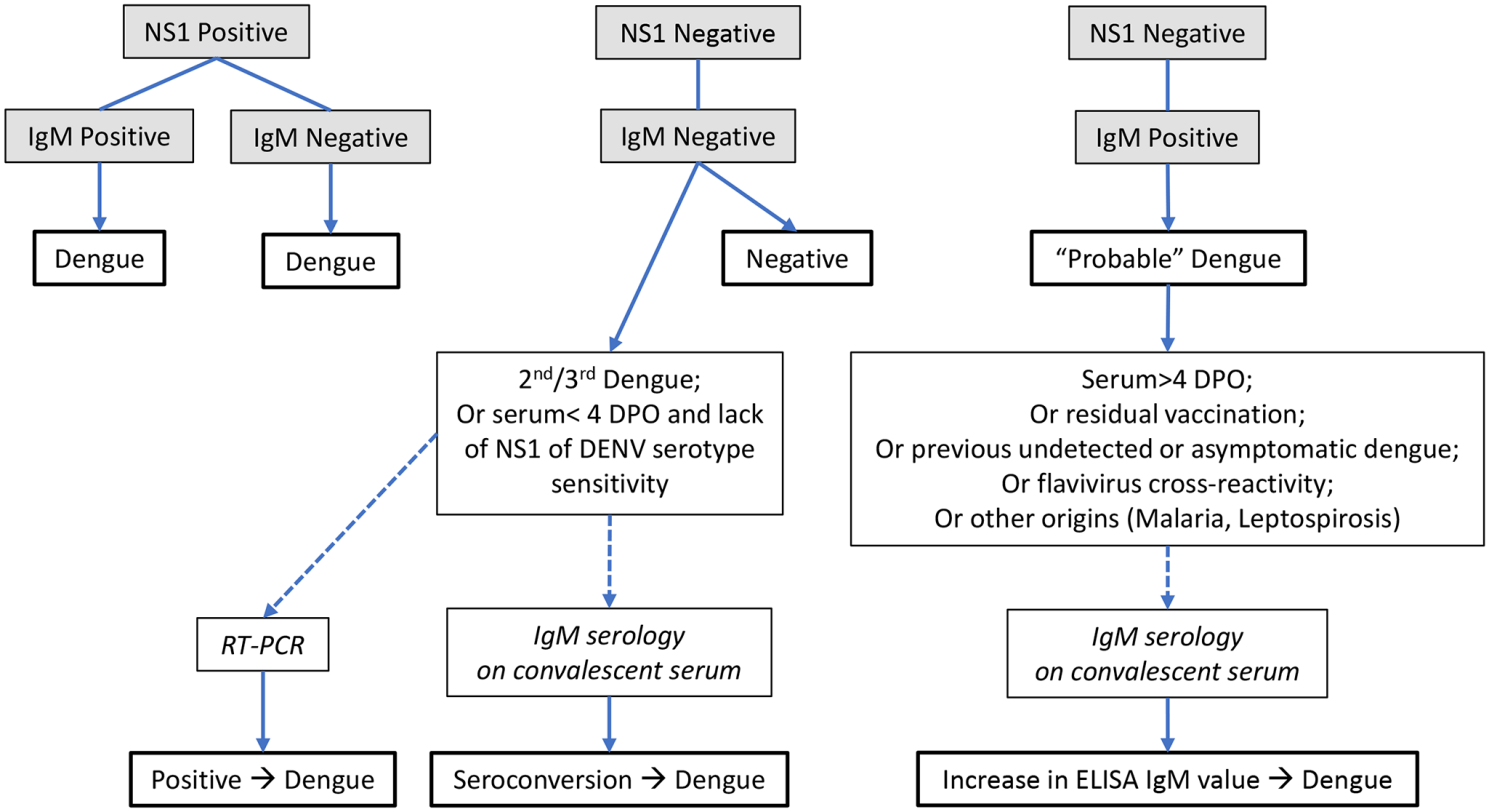
Proposed algorithm of dengue diagnostic testing in acute serum of patients suspected of dengue.

Fig 1 indicates an algorithm of dengue diagnostic testing in acute sera of patients suspected of dengue disease, using a combination of IgM and NS1 ELISAs. Positive NS1 ELISA usually offers an unambiguous result of current dengue infection when sera are collected during the acute phase of infection (Table 1; Fig 1). However, ZIKV, which is endemic in Southeast Asia and the Americas since 2015, cocirculates with DENV and has recently shown cross-reactivity with DENV NS1 [13], leading to potential false positive result in dengue NS1 ELISA [14]. As different kits for DENV NS1 testing utilize anti-NS1 monoclonal antibodies with different specificities, they should be more carefully evaluated for possible cross-reactivity between DENV and ZIKV.

In the majority of cases, negative IgM and NS1 antigen ELISAs exclude a dengue infection. However, in secondary or tertiary dengue infection, IgM may be undetectable [3,6], and false negative NS1 may be related to the low sensitivity of the assay for some DENV serotypes/genotypes due to the presence of NS1-antibody immune complexes [8,15]. Negative NS1 ELISA results on acute samples should be tested by RT-PCR for DENV (Fig 1). Moreover, RT-PCR is generally more specific and sensitive than NS1 ELISA and determines the infecting viral serotype, which can inform vaccine developers of breakthrough infections by a specific serotype. Alternatively, IgM ELISA should be tested on a convalescent serum (Fig 1). A dengue infection is confirmed only by seroconversion.

“Probable” dengue cases are defined as NS1 ELISA negative when sera are collected ≥5 DPO and IgM ELISA positive or low/borderline positive (Table 1; Fig 1). However, a >2-fold increase in IgM titers calculated on a second serum specimen is suggestive of an acute infection or seroconversion. False positive IgM may result from individuals vaccinated with CYD-TDV at least 2 months earlier, which may interfere with IgM results and are considered in the diagnosis of “probable” dengue [4] (Table 1; Fig 1). Undetected and recent asymptomatic dengue or other cocirculating flaviviruses may induce IgM and should also be considered in the analysis of “probable” dengue [3,5,16]. Dengue IgM tests may also show false positives in sera from patients infected with other pathogens such as *Plasmodium* parasite or *Leptospiroris* bacteria [6] (Fig 1). False IgM positivity impacted by heterophilic antibodies in antibody capture ELISA could be mitigated by using background subtraction procedure and in-house algorithm definition [17]. In addition, the cutoff for positive values could be modified by using a receiver operator curve to identify the proper cutoff value for the population studied. Alternatively, serial dilution of the specimen could provide the optimal dilution, which represents the dynamic range of the curve [6]. When possible, a second convalescent serum specimen should be obtained and tested for IgM when there is an ambiguous result in the IgM ELISA.

The diagnosis of DENV is a complex process made even more delicate after the implementation of vaccination against this virus. The results of the laboratory must be interpreted with the support of several pieces of information: in particular, (a) the age of patient, (b) the sampling date post-onset of illness, (c) clinical signs and symptoms and hematological parameters of the patient (e.g., leukopenia and thrombocytopenia) [3], (d) the history of flavivirus infection/vaccination, and (e) the epidemiological context in areas where other flaviviruses cocirculate.

## Conclusions

To assess the overall benefits of vaccination in high burden countries for dengue, long-term surveillance will require a robust, affordable, and user-friendly diagnostic algorithm to detect suspected dengue cases. Based on previous experience, there is a limitation of detecting solely anti-DENV IgM antibody responses in a single acute serum sample for individuals who have been vaccinated within 2 months. A more reliable diagnosis for detecting an acute infection is a combination of NS1 and IgM ELISAs. Only a few published studies have used the combination of anti-DENV IgM ELISA and NS1 ELISA in the context of a “probable” dengue case. These were retrospective studies using virologically confirmed sera by RT-PCR and NS1 ELISA and tested for IgM and IgG using paired specimen of acute and convalescent sera, respectively [4,12]. Thus, in order to address the sensitivity of the combination of IgM and NS1 ELISAs in a population in which the majority of individuals are seropositive for DENV and/or other flaviviruses (e.g., ZIKV, JEV, or YFV) and vaccinated with CYD-TDV, a prospective study is required.

Since a large variability in the results of the diagnostics may occur between different kits and laboratories worldwide, future phase IV studies of CYD-TDV vaccine effectiveness should use the proposed combined diagnostic testing with the same validated and standardized kits/protocols and international guidelines when designing sentinel sites to ensure quality assurance and to enhance data sharing and comparability across regions [10].
